# Identification of Conserved and Novel MicroRNAs in Blueberry

**DOI:** 10.3389/fpls.2017.01155

**Published:** 2017-06-30

**Authors:** Junyang Yue, Xiaohui Lu, Huan Zhang, Jiao Ge, Xueling Gao, Yongsheng Liu

**Affiliations:** ^1^School of Tea and Food Science, Anhui Agricultural UniversityHefei, China; ^2^College of Food Science and Engineering, Hefei University of TechnologyHefei, China; ^3^Hefei National Laboratory for Physical Sciences at Microscale, School of Life Sciences, University of Science and Technology of ChinaHefei, China

**Keywords:** *Vaccinium* sect. Cyanococcus, blueberry, miRNA, high-throughput sequencing, gene expression

## Abstract

MicroRNAs (miRNAs) are a class of small endogenous RNAs that play important regulatory roles in cells by negatively affecting gene expression at both transcriptional and post-transcriptional levels. There have been extensive studies aiming to identify miRNAs and to elucidate their functions in various plant species. In the present study, we employed the high-throughput sequencing technology to profile miRNAs in blueberry fruits. A total of 9,992,446 small RNA tags with sizes ranged from 18 to 30 nt were obtained, indicating that blueberry fruits have a large and diverse small RNA population. Bioinformatic analysis identified 412 conserved miRNAs belonging to 29 families, and 35 predicted novel miRNAs that are likely to be unique to blueberries. Among them, expression profiles of five conserved miRNAs were validated by stem loop qRT-PCR. Furthermore, the potential target genes of conserved and novel miRNAs were predicted and subjected to Gene Ontology (GO) annotation. Enrichment analysis of the GO-represented biological processes and molecular functions revealed that these target genes were potentially involved in a wide range of metabolic pathways and developmental processes. Particularly, anthocyanin biosynthesis has been predicted to be directly or indirectly regulated by diverse miRNA families. This study is the first report on genome-wide miRNA profile analysis in blueberry and it provides a useful resource for further elucidation of the functional roles of miRNAs during fruit development and ripening.

## Introduction

*Vaccinium* sect. Cyanococcus is a subgenus within the Ericaceae and well known as blueberry. It is native of North America although nowadays is widely distributed around the globe (Rimando et al., 2004). Through decades of selection from the wild germplasm, several economically important horticultural species have been successfully domesticated, including *V. angustifolium, V. corymbosum* and *V. ashei*. When the blueberry fruits ripen, the flesh turns from pale greenish to dark purple, due to an abundant accumulation of polyphenolic anthocyanin pigments (Stevenson and Scalzo, 2012).

Nowadays, blueberry has become an economically and nutritionally important fresh fruit (Wu et al., 2004). Accumulating studies have shown that blueberry contains much more abundant antioxidants compared to the conventional staple fruits, such as grape, apple, and tomato (Wolfe and Liu, 2007; Nile and Park, 2014). Dietary consumption of these antioxidant metabolites had been demonstrated to be very beneficial to human health and could provide effective protection against diseases caused by free radicals (Zheng and Wang, 2003). Particularly, the compositions of antioxidants in blueberries are a group of carotenoids, anthocyanins, vitamins and phenolic compounds. Among them, the anthocyanins have been shown to execute essential functions in preventing or alleviating serious disorders, such as coronary heart disease (Neto, 2007), diabetes (Prior et al., 2008), cancer (Faria et al., 2010), or even slowing down the effects of aging (Grace et al., 2009). Therefore, it is important to better understand the molecular mechanisms involved in the biosynthesis and accumulation of antioxidant metabolites in blueberries.

Genomic and transcriptomic data have been recently released for blueberries (Zifkin et al., 2012; Bian et al., 2014). As of April 2016, 333 nucleotide sequences and 19,908 expressed sequence tags (ESTs) were deposited in the GenBank database. The ESTs available could be employed to investigate wide varieties of genetic characteristics and identify candidate genes associated with agriculturally important traits. More recently, a draft genome assembly of a diploid northern highbush blueberry was generated using the high-throughput sequencing technology, which could provide a general and comprehensive platform for gene expression profiling studies (Gupta et al., 2015).

miRNAs, a class of endogenous non-coding regulatory RNAs with stem-loop structures, are originated from long primary transcripts (pri-miRNAs) and finally processed into 19–25 nucleotides in size (Axtell, 2013). They have been demonstrated to regulate gene expression either through direct cleavage of transcripts or translational repression at post-transcriptional level, or in some case, by methylation at transcriptional level in plants (Brodersen et al., 2008). In addition, pri-miRNAs have recently been reported to harbor short open reading frames (ORFs) and encode miRNA-encoded peptides (miPEPs), which could enhance the expression of their corresponding miRNAs in turn (Couzigou et al., 2015; Lauressergues et al., 2015; Lv et al., 2016). However, no genome-scale expression data is available for miRNA sequences as well as their expression patterns in blueberries. To broaden the scope of genetic information buried in the blueberry genome, we performed the ^∗∗∗^first global analysis of small RNA transcriptome from ripening fruits using the high-throughput Illumina technology in the present study. The results we obtained could help further elucidating their tissue-specific functions and understanding the mechanisms of anthocyanin biosynthesis from a new perspective.

## Materials and Methods

### Plant Material and Sample Collection

Blueberry (*V. ashei*) was grown under natural conditions in a field nursery at Hefei, Anhui province, People’s Republic of China. Ripening fruits with 50 days after full bloom were collected from 5-year-old plants, frozen immediately in liquid nitrogen and stored at -80°C until further use. The location of the field nursery is not privately owned or protected in any way.

### Small RNA Library Preparation and Sequencing

Total RNAs were extracted from blueberry fruits using the TRIzol reagent (Invitrogen, United States). RNA quality and quantity were evaluated using an Agilent 2100 bioanalyzer (Agilent Technologies, CA). Only the samples with a 28S/18S ratio of nearly 2.0 were subjected to 15% (w/v) denaturing polyacrylamide gel electrophoresis (PAGE) and the small RNA fragments of 18–28 nt were isolated and purified. Next, the small RNA molecules were sequentially ligated to 5′ and 3′ adaptors, and then converted to cDNA by reverse transcription polymerase chain reaction (RT-PCR) amplifications. Finally, approximately 20 μg of the RT-PCR products were directly sequenced using Illumina Genome Analyzer according to the manufacturer’s protocols. The sequencing data have been deposited in NCBI’s Gene Expression Omnibus (Edgar et al., 2002) and are accessible through GEO Series accession number GSE94411.^1^

### Small RNA Analysis

After removing the 5′ adaptor sequences (GTTCAGAGTTCTACAGTCCGACGATC), trimming the 3′ acceptor sequences (TCGTATGCCGTCTTCTGCTTG), filtering those low quality reads with a quality value (Q) less than 10 (the quality value was calculated as following: Q = ASCII character code – 64) and cleaning up contaminated reads (Li et al., 2008), we finally obtained the clean reads. The occurrence of each detected read was counted as tags and the length distribution of these unique tags was analyzed. Then, all tags were mapped to the blueberry cDNA sequences released recently (Gupta et al., 2015)^2^ using the BLASTN program with an *E*-value of 0.1. Any matched tags were considered as coding sequences and excluded. To identify conserved miRNAs in blueberries, the remaining tags were aligned with known miRNA precursors and mature miRNAs present in the miRBase database (Kozomara and Griffiths-Jones, 2014; Release 21.0)^3^ using the BLASTN program (*E*-value = 0.1). Tags with perfect match or no more than two mismatches were retained for further analysis, whereas the unmatched tags were then matched to non-coding RNAs (rRNA, tRNA, snRNA, and snoRNA) available in the Rfam database (Nawrocki et al., 2014; Release 12.1)^4^ for classification. Tags not matched in the above databases were defined as unclassified tags.

### Prediction of Novel miRNAs

The unclassified tags consisting of at least 10 reads were retained and processed for novel miRNA prediction. The cutoff of 10 reads was chosen to reduce false positives. First of all, these tags were mapped to the blueberry EST sequences with a perfect match (*E*-value = 0.1). Then, any matched EST sequences were selected as the potential miRNA precursors. Next, the secondary structures of these potential miRNA precursors were predicted by the RNAfold WebServer program (Hofacker, 2004).^5^ Finally, the putatively novel miRNAs were identified based on the following rules: (1) the minimal length of miRNA sequences is 18 nt, (2) the maximal length of miRNA sequences is 30 nt, (3) the maximal free energy (MFE) allowed for each miRNA precursor is -18 kcal/mol, (4) the maximal bulge of miRNA and miRNA^∗^ is 4, (5) no more than 3 adjacent mismatches between miRNA and miRNA^∗^, (6) the miRNA and miRNA^∗^ should be from the same EST sequence, and (7) 30–70% of CG content in the putative miRNA precursors (Zhang et al., 2006a, 2012; Meyers et al., 2008). To further confirm blueberry-specific novel miRNAs, the obtained sequences above were additionally mapped to the cDNA sequences of Arabidopsis (Berardini et al., 2015), cranberry (Polashock et al., 2014), tomato (Tomato Genome Consortium, 2012), grape (Jaillon et al., 2007) and kiwifruit (Yue et al., 2015a) using the BLASTN program (*E*-value = 0.1). Only the unmatched sequences were considered as putatively novel miRNAs in blueberries.

### Prediction of miRNA Targets

The putative targets of conserved and novel miRNAs were predicted by aligning the miRNA sequences with the blueberry cDNA sequences using the psRNATarget online program (Dai and Zhao, 2011).^6^ The stringent criteria with the following rules were employed: (1) perfect match at the seed region (in positions 2–8 nt from the 5′ end of miRNA sequences), (2) the maximum expectation is 3, (3) the length for complementarity scoring is 20, and (4) the allowed maximum energy to unpair the target site (UPE) is 25. Additionally, whether the matches in positions 9–11 nt from the 5′ end of miRNA sequences is used to estimate the pattern of regulatory mechanisms.

### GO Analysis of miRNA Target Genes

The predicted target genes of miRNAs were assigned to molecular function and biological process categories according to GO annotation using the Blast2GO pipeline (Götz et al., 2008). The enrichment analysis of each GO term was performed using the Hypergeometric test. Here, we defined *N* as total number of annotated GO terms in the whole genome, *M* as total number of a specific GO term in the whole genome, *n* as total number of GO terms in the predicted miRNA target genes, *k* as total number of a specific GO term in the predicted miRNA target genes, *d* as the number of different GO terms in the predicted miRNA target genes, and *r* as the rank of each *P*-value from small to large order. Then, the *P*-value was calculated and subsequently assigned by Benjamini–Hochberg adjustment as follows:

$$\begin{array}{c} \;f(k;n,m,N)=\frac{\left(\overset{m}{\underset{k}{}}\right)\left(\overset{N-m}{\underset{n-k}{}}\right)}{\left(\overset{N}{\underset{n}{}}\right)}\\ \;P-value=1-f(k;n,m,N)\\ \mathrm{Adjusted}\;P-value =P-value \end{array}$$

Any GO terms with an adjusted *P*-value less than 0.05 were regarded as the enriched ones.

### Reverse Transcription and Quantitative Real-time PCR Analysis of miRNAs

The expression levels of miRNAs in ripening fruits were validated by quantitative real-time PCR (qRT-PCR) using an Applied Biosystems StepOne^TM^ Real-Time PCR System (Applied Biosystems, Waltham, MA, United States) and a FastStart Universal SYBR Green Master (Roche, Switzerland). The sequences of miRNAs were reverse transcribed to cDNAs with stem-loop primes by using the PrimeScript RT Reagent Kit and gDNA Eraser (Takara, Dalian, China). Their primers were listed in Supplementary Table S1. The reactions were performed in a 48-well optical plate using the following conditions: an initial polymerase activation step for 10 min at 95°C, 40 cycles of 15 s at 95°C for denaturation, 1 min at 60°C for annealing and elongation. After reaction, the threshold cycle (Ct), defined as the fractional cycle number at which the fluorescence passed a fixed threshold, was determined using the default threshold settings. The expression level of snRNA U6 was used as an internal reference.

## Results

### Sequence Analysis of Short RNAs

**FIGURE 1:**
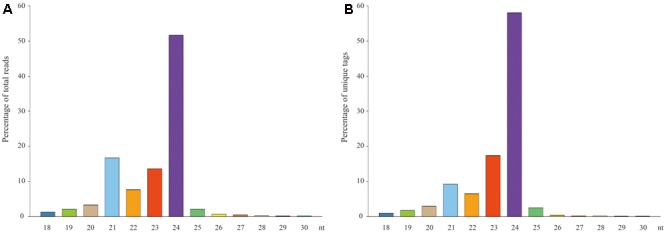
Distribution of small RNAs with different sequence length according to their total reads and unique tags. **(A)** Proportion analysis of total reads, **(B)** Proportion analysis of unique tags.

In order to obtain a comprehensive view of the small RNAs expressed in blueberries, we have performed the Illumina technology for deep sequencing a small RNA library derived from ripening fruits. A total of 21,721,825 raw reads were collected by sequencing. After removing the adaptors, cleaning up contaminations and filtering out low quality reads, 20,909,994 clean reads with sizes ranged from 18 to 30 nt were obtained (**Table 1**). Among them, a majority (95.0%) were 20–25 nt and the most abundant were 24 (51.6%) nt in length, followed by 21 (16.7%) and 23 (13.6%) nt (**Figure 1A**). Furthermore, these 20,909,994 clean reads were represented by 9,992,446 unique tags, of which 36 were sequenced more than 10,000 times, whereas 7,012,938 (70.2%) and 1,752,844 (17.5%) were present only one and two times, respectively. Size distribution analysis of these unique tags showed that the predominant sequences were 24 nt in length, accounting for approximately 58.1% of the total tags (**Figure 1B**). A slight difference exists in that the second largest group of tags was 23 nt (17.3%) and the third was 21 nt (9.2%). These results suggest that three groups of 24, 23, and 21 nt small RNAs are the dominant classes of small non-coding RNAs in blueberry fruits, which is similar to the distribution patterns analyzed from tomato (Wu et al., 2016), Arabidopsis (Lu et al., 2005), rice (Morin et al., 2008), and maize (Wang et al., 2011).

**Table 1:** Statistics of sequencing reads.

Type	Count	%
Total raw reads	21,721,825	
Low quality reads	24,375	0.112
High quality reads	21,697,450	99.888
3′ adapter null	204,342	0.941
Insert null	38,469	0.177
5′ adapter contaminants	18,286	0.084
Smaller than 18 nt	502,984	2.316
Larger than 30 nt	22,915	0.105
Poly A	460	0.002
Clean reads	20,909,994	96.263

### Identification of Conserved miRNAs in Plants

Among the 9,992,446 unique tags, 3,006,253 tags were mapped to the cDNA sequences in the blueberry genome, whereas a total of 121,459 tags were annotated as several distinct categories of non-coding RNAs in the Rfam database, such as rRNA, tRNA, snRNAs and snoRNAs (**Table 2**). In comparison, 412 unique tags corresponding to 11,898 reads were matched to known mature miRNAs collected from plants in the miRBase database and finally reserved as conserved miRNAs in blueberries with manual check.

**Table 2:** Categorization of unique tags.

Category	Count	%
All unique tags	9,992,446	
Map to cDNA	3,006,253	30.085
rRNA	59,818	0.599
tRNA	15,303	0.153
snoRNA	6,958	0.07
snRNA	272	0.003
Others in Rfam	39,108	0.391
Match in miRBase^∗^	412	0.004
Unclassified	6,864,322	68.695

*^∗^Only the mature miRNAs matched from plants were identified.*

The length of these conserved miRNAs identified varies from 18 to 29 nt. Among them, the sequences of 20–24 nt accounted for approximately 86.89%, which is consistent with the main size variation of miRNAs derived from Dicer digestion products (Borges and Martienssen, 2015). Further classification of individual sequence length revealed that 21-nt miRNAs were the major type accounting for 164, which is identical to the previous reports from other plant species (Rogers and Chen, 2013; Wu et al., 2016). The complete lists of miRNA sequences, sequence length and read counts were shown in Supplementary Table S2.

**FIGURE 2:**
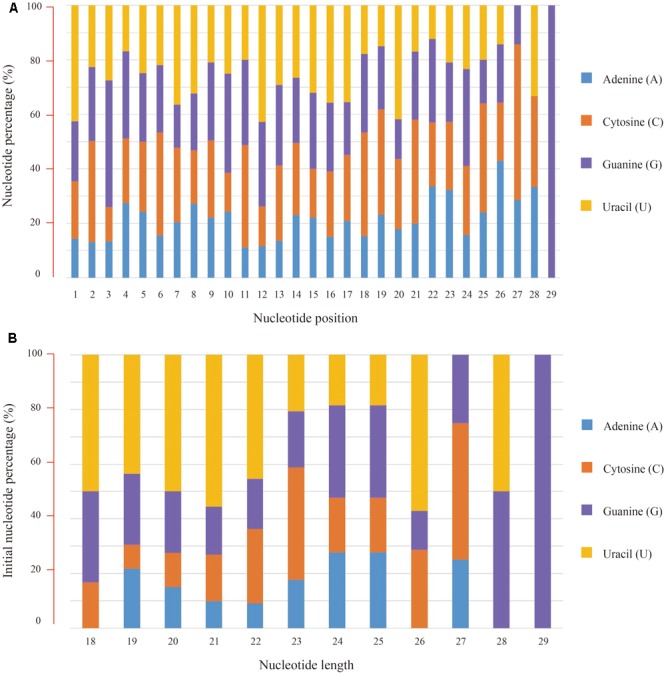
Nucleotide composition of the conserved miRNAs and their bias positions. **(A)** Nucleotide composition and bias for individual positions from the 5′ end of miRNA sequences, **(B)** Nucleotide composition and bias for the first position of miRNAs with different length.

Subsequently, nucleotide composition of these conserved miRNAs was calculated and the overall percentages of individual bases were 19.33% for adenine (A), 26.59% for cytosine (C), 26.77% for guanine (G) and 27.31% for uracil (U), respectively. Position-specific base analysis showed that U (42.48%) was the preferred initial base toward the 5′ end of these conserved miRNA sequences. C (46.60%) was found to dominate the third base position, while again the U (42.72%) was abundant at the 12th position (**Figure 2A**). Further analysis of the initial bases in miRNAs with different nucleotide length revealed that the “U” bias was predominately present in 18–22 nt sequences (**Figure 2B**).

**FIGURE 3:**
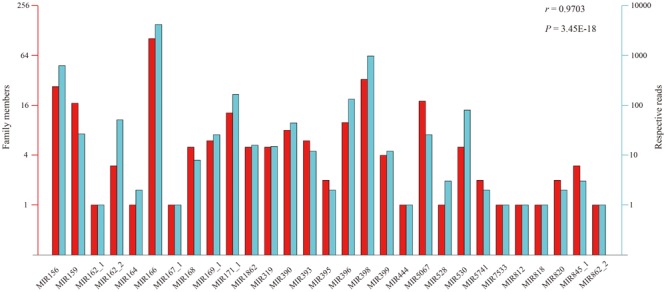
The correlation of family members and their respective reads within each family.

According to the constructed miRNA families in the Rfam database, the above 412 conserved miRNAs could be grouped into 29 putative families (**Figure 3**). However, large variation in number of unique tags existed among the individual miRNA families. Six miRNA families (MIR166, MIR398, MIR156, MIR5067, MIR159, and MIR171_1) had more than 10 members, wherein the MIR166 family was the largest, containing 99 members. By contrast, merely one member occurred in the MIR167_1, MIR164, MIR162_1, MIR818, MIR812, MIR444, MIR528, MIR862_2, and MIR7533 families. Likewise, the read number of these miRNA families also varied to a large extent ranging from 1 to 4,215, and the MIR166 family possessed the largest number of reads, followed by the MIR398 and MIR156 families, with 989 and 637 reads, respectively (**Figure 3**). Not surprisingly, a significant positive correlation of 0.9703 (Pearson test, *P*-value = 3.45 × 10^-18^) was observed between the number of family members and their corresponding reads among the 29 miRNA families.

**FIGURE 4:**
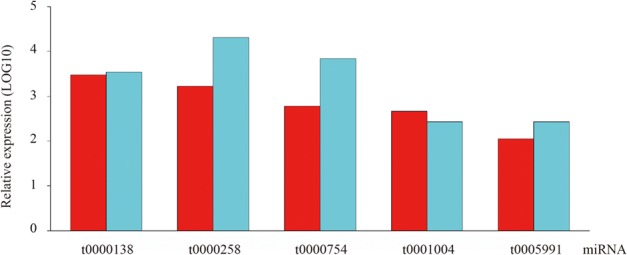
The existence and expression level of five random selected conserved miRNAs were confirmed by qRT-PCR in ripening fruits of blueberry.

To confirm the high-throughput sequencing data, qRT-PCR was employed as an alternative method due to its sensitivity, specificity and rapidity in the current study. Specifically, five conserved miRNAs were randomly chosen for quantification and the result showed their expression patterns are consistent with those obtained from high-throughput sequencing technology (**Figure 4**).

### Identification of Novel miRNAs in Blueberries

To identify the unique miRNAs potentially occurred in blueberry fruits, the unclassified tags were further subjected to predicting novel miRNA candidates using strict criteria. Consequently, 35 novel miRNAs were identified and their characteristic hairpin secondary structures were deduced (Supplementary Table S3). All these identified novel miRNAs had at least one perfect match to the EST sequences and varied in size ranging from 18 to 24 nt. According to previous reports, the minimal folding free energy index (MFEI) of a given miRNA precursor was more likely to be greater than the values of tRNAs (∼0.64), rRNAs (∼0.59), mRNAs (∼0.66), and even random sequences (Bonnet et al., 2004; Zhang et al., 2006b). Therefore, this important feature could be employed to distinguish the miRNA precursors from other types of RNAs and identify unique miRNA sequences. In the current study, the MFEI value of predicted novel miRNA precursor sequences ranged from 0.50 to 1.08 with an average of 0.76.

### Prediction of miPEPs in Blueberries

To demonstrate whether the pri-miRNAs of the identified miRNAs potentially encode miPEPs, we analyzed the upstream sequences of the available pri-miRNA datasets from blueberries. As a result, most of the pri-miRNAs were predicted to contain one or more putative ORF, with a size ranging from 4 to 75 amino acid residues (Supplementary Table S4).

### Prediction of miRNA Targets

In order to elucidate the biological functions of the identified miRNAs, their putative target genes were predicted using the psRNATarget program and subsequently analyzed based on the GO annotation (Supplementary Table S5). Here, all the identified conserved and novel miRNAs are found to have potential target candidates. GO enrichment analysis revealed that the target genes of conserved miRNAs appeared to be enriched in seed maturation (GO:0010431), while the target genes of novel miRNAs were significantly related to response to carbon dioxide (GO:0010037), cellular ion homeostasis (GO:0006873), zinc II ion transport (GO:0006829), regulation of nucleobase-containing compound metabolic process (GO:0019219), and meiosis I (GO:0007127) (Supplementary Tables S6, S7).

In addition, the target gene number of individual miRNAs varies greatly from 3 to 128. For instance, t9199089 (belongs to the MIR159 family) tops the list with 128 targets, followed by t0107896 and t0177138 (both belong to the MIR396 family) which have 100 and 84 targets, respectively. From an overall perspective of the miRNAs with more than 50 predicted targets, many transcription factors and enzymes were predicted to be the target proteins, such as R2R3 MYB transcription factor, cyclin-dependent protein kinase, COP1 homolog and squamosa promoter binding-like protein (Supplementary Table S8). GO analysis of these proteins showed an enrichment of potassium ion import (GO:0010107), pyrimidine nucleobase biosynthetic process (GO:0019856) and stomatal movement (GO:0010118) in the biological process category, whereas only dihydroorotase activity (GO:0004151) in the molecular function category (Supplementary Table S9).

## Discussion

Fruit development and ripening is a multi-faceted but tightly regulated process. Up to date, many critical molecules responsible for fruit enlargement, texture alteration and nutrients accumulation have been identified based on both forward and reverse genetic approaches (Yue et al., 2015b). Recently, high-throughput sequencing technology resulted in a sharp increase in the magnitude of genome information and gene expression profiles. Many studies have shown that miRNAs possess important regulatory roles in the process of fruit development and ripening (Zeng et al., 2015; Šurbanovski et al., 2016). Firstly, several expression profile studies indicated that numerous miRNAs are exclusively or preferentially expressed in fruits (Liu et al., 2014). Secondly, distinct miRNA expression patterns manifest at different developmental stages (Wu et al., 2014). And last, fruit growth could be disrupted with conditional miRNA transgene (Xian et al., 2014), which provides direct evidence for their regulatory functions during fruit development and ripening. In the present study, we employed the Illumina sequencing technology to generate a genome-wide small RNA transcriptome in blueberries, aiming to understand their putative roles particularly in the biosynthesis and accumulation of antioxidant metabolites.

Our study identified 412 conserved miRNAs with a distribution ranging in 29 families. Among these conserved families, MIR166 was most abundant and predicted to target 796 genes. So far, the MIR166 family was found in all plant lineages, indicating that miRNA-mediated interactions possess an essential function across the plant kingdom and have existed for at least 425 million years (Zhang et al., 2005). Experimental studies have also demonstrated that MIR166 was ubiquitously expressed and fairly abundant in all the analyzed tissues, which suggests that it is required in all cell types to carry out housekeeping functions in plants (Barik et al., 2014). Here in this study, various transcription factors including four class III homeodomain leucine zipper family (ZIP) transcripts, four ethylene response factor (ERF) transcripts, six squamosa promoter binding-like protein (SPB/SPL) transcripts, four MADS transcripts, six basic helix-loop-helix (bHLH) transcripts, two R2R3 MYB transcripts and six auxin response factor (ARF) transcripts were predicted as candidate targets of the MIR166 family (Supplementary Table S10). Some of them have been identified to participate in regulatory pathways of anthocyanin biosynthesis, such as SPL in Arabidopsis (Gou et al., 2011), MYB in tomato (Jia et al., 2015), and bHLH in apple (Feng et al., 2013). Of course, the tremendous targets predicted from *in silico* data might be overstated based on existing hypothesis, and the actual interactions between a given miRNA as well as its targets still need further experimental validations.

While a single type of miRNA can bind many gene products, multiple miRNAs can potentially regulate a single gene as well. On the basis of prediction, SPL transcripts were identified as targets of 16 different miRNA families. Recent studies have revealed that SPL acts as a pleiotropic regulator of plant development and could negatively regulate anthocyanin accumulation by preventing the expression of anthocyanin biosynthetic genes (Wang et al., 2009). Therefore, reducing the expression of SPL would promote the accumulation of anthocyanins. Up to date, the MIR156 has been confirmed to positively regulate the accumulation of anthocyanins through the directed cleavage of SPL transcripts in Arabidopsis and rice (Gou et al., 2011; Xie et al., 2012). In considering that most of the miRNA targets are conserved across plants (Adai et al., 2005), we have reason to believe that the high level of MIR156 transcripts detected would also accelerate anthocyanin accumulation in ripening blueberry fruit. Additionally, both MIR828 and MIR858 have been shown to act as negative regulators of anthocyanin biosynthesis by the cleavage of MYB transcription factors (Luo et al., 2012; Jia et al., 2015), but their homologs were not detectible in our study (Supplementary Table S10), consequently suggesting an enhanced anthocyanin synthesis in blueberries. Therefore, the potential roles of these miRNAs involved in anthocyanin biosynthesis are worth further exploring, and their expression patterns in a series of developmental stages are expected to be systematically detected and compared.

**FIGURE 5:**
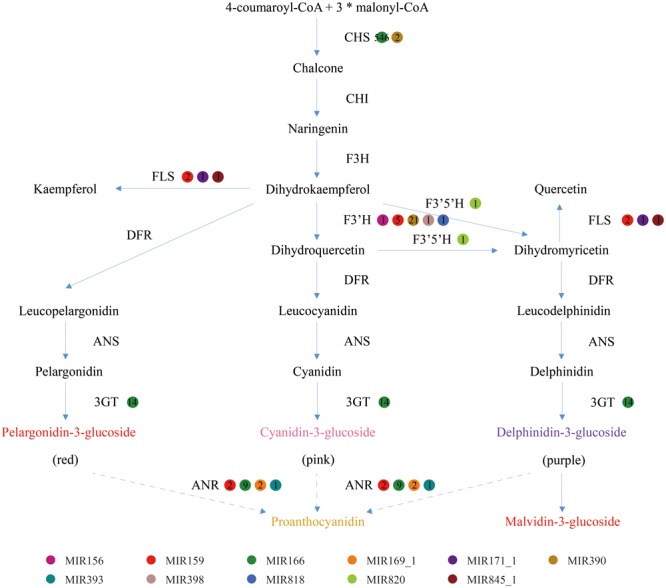
The enzyme-coding structural genes involved in anthocyanin biosynthetic pathway associated with the putative regulated miRNAs and their expression levels. CHS, chalcone synthase; CHI, chalcone isomerase; F3H, flavanone 3-hydroxylase; F3′H, flavonoid 3′-hydroxylase; F3′5′H, flavonoid-3′, 5′-hydroxylase; DFR, dihydroflavonol 4-reductase; ANS, anthocyanidin synthase; 3GT, UDP-glucose-flavonoid 3-*O*-glucosyltransferase; and FLS, flavonol synthase.

Generally, the genes involved in anthocyanin biosynthesis have been extensively characterized (Gandía-Herrero and García-Carmona, 2013) and were divided into two categories (Holton and Cornish, 1995): structural genes which encode anthocyanin biosynthetic enzymes and regulatory genes that control the expression of structural genes. Apart from regulating anthocyanin biosynthesis by cleavage of transcription factors to affect the expression of structural genes, miRNAs could also directly inhibit the expression of structural genes. As shown in **Figure 5**, the structural genes participating in anthocyanin biosynthesis pathways include chalcone synthase (CHS), chalcone isomerase (CHI), flavanone 3-hydroxylase (F3H), flavonoid 3′-hydroxylase (F3′H), flavonoid-3′,5′-hydroxylase (F3′5′H), flavonol synthase (FLS), dihydroflavonol 4-reductase (DFR), anthocyanidin synthase (ANS) and UDP-glucose-flavonoid 3-*O*-glucosyltransferase (3GT). In the present study, five structural genes were found to be targeted by diverse miRNAs with different expression levels, while four genes were predicted not to be targeted by any miRNA families (**Table 3**).

**Table 3:** Structural genes involved in anthocyanin biosynthetic pathway and their putative regulated miRNA families.

miRNA family	Target genes	Gene ID	Total reads
MIR156	F3′H	gene.g643.t1.1	1
MIR159	ANR	CUFF.44100.2, CUFF.44100.1	2
	F3′H	CUFF.55591.1	5
	FLS	CUFF.2063.1	2
MIR166	3GT	CUFF.38623.1	14
	ANR	CUFF.20635.1, CUFF.9281.1	9
	CHS	CUFF.20573.1, CUFF.47176.1	546
MIR169_1	ANR	CUFF.11112.1	2
MIR171_1	FLS	CUFF.11151.1	1
MIR390	CHS	CUFF.9485.1	2
	F3′H	CUFF.35973.1, 50629_g.1	21
MIR393	ANR	CUFF.26139.1	1
MIR398	F3′H	CUFF.34750.1	1
MIR818	F3′H	gene.g26577.t1.1	1
MIR820	F3′5′H	CUFF.21569.1	1
MIR845_1	FLS	CUFF.26171.1	1

Although the anthocyanin composition of blueberries and their accumulation levels fluctuate with species, cultivars and growth conditions, delphinidin and malvidin have been found to be the most predominant in many species, especially the highbush blueberries (Kalt et al., 1999). The small amount of cyanidin, a major compound of anthocyanins, could be interpreted by the destructive function of miRNAs toward the corresponding structural genes. Actually, our study found F3′H transcripts were putatively regulated by five miRNA families that could cause an inhibitory action in metabolic pathways of cyanidin (**Figure 5**). Consequently, large proportion of substrate precursor could be allocated to produce delphinidin/malvidin, leading to an increased delphinidin/malvidin accumulation. In addition, four miRNA families were implicated in targeting anthocyanidin reductase (ANR), an enzyme that converts anthocyanidins to proanthocyanidins for destruction, probably providing a favorable condition for anthocyanin accumulation. The expression levels of the above miRNAs and their potential target genes could suggest a coordinated post-transcriptional regulatory network during fruit ripening. However, it seems hardly understanding why the miRNAs that target CHS transcripts displayed an abundant expression level and the reason still need further studies. To reduce systemic errors in data analysis, we will keep updating the miRNA gene annotation as new version of the blueberry genome released.

In summary, we have investigated the conserved and novel miRNAs from ripening fruits in blueberries by using the high-throughput sequencing technology for the first time. Stem-loop RT-PCR experiment was used to confirm the expression of these miRNAs. Furthermore, the GO annotation and pathway analysis for predicted targets have implicated the putative roles of the abundant miRNAs during the processes of fruit ripening and nutrient accumulation. Notably, this study provides a useful resource for further elucidation of regulatory functions of miRNAs in anthocyanin biosynthesis and may facilitate the design of genetically modifying plants.

## Author Contributions

JY, XG, and YL planned the project and designed the experiments. XL and HZ performed the experimental work. JY, XG, YL, and JG participated in the discussions, produced the first draft and the revised manuscript.

## Conflict of Interest Statement

The authors declare that the research was conducted in the absence of any commercial or financial relationships that could be construed as a potential conflict of interest.

## Footnotes

1 https://www.ncbi.nlm.nih.gov/geo/query/acc.cgi?acc=GSE94411

2 https://bitbucket.org/lorainelab/blueberrygenome

3 http://www.mirbase.org/

4 http://rfam.xfam.org/

5 http://rna.tbi.univie.ac.at//cgi-bin/RNAWebSuite/RNAfold.cgi

6 http://plantgrn.noble.org/psRNATarget/
